# An ontological approach to identifying cases of chronic kidney disease from routine primary care data: a cross-sectional study

**DOI:** 10.1186/s12882-018-0882-9

**Published:** 2018-04-10

**Authors:** Nicholas I. Cole, Harshana Liyanage, Rebecca J. Suckling, Pauline A. Swift, Hugh Gallagher, Rachel Byford, John Williams, Shankar Kumar, Simon de Lusignan

**Affiliations:** 1 South West Thames Renal Department, St Helier Hospital, Wrythe Lane, Carshalton, UK; 2 Department of Clinical and Experimental Medicine, University of Surrey, Guildford, UK

**Keywords:** Chronic kidney disease, Epidemiology, Ontology, eGFR, Proteinuria

## Abstract

**Background:**

Accurately identifying cases of chronic kidney disease (CKD) from primary care data facilitates the management of patients, and is vital for surveillance and research purposes. Ontologies provide a systematic and transparent basis for clinical case definition and can be used to identify clinical codes relevant to all aspects of CKD care and its diagnosis.

**Methods:**

We used routinely collected primary care data from the Royal College of General Practitioners Research and Surveillance Centre. A domain ontology was created and presented in Ontology Web Language (OWL). The identification and staging of CKD was then carried out using two parallel approaches: (1) clinical coding consistent with a diagnosis of CKD; (2) laboratory-confirmed CKD, based on estimated glomerular filtration rate (eGFR) or the presence of proteinuria.

**Results:**

The study cohort comprised of 1.2 million individuals aged 18 years and over. 78,153 (6.4%) of the population had CKD on the basis of an eGFR of < 60 mL/min/1.73m^2^, and a further 7366 (0.6%) individuals were identified as having CKD due to proteinuria. 19,504 (1.6%) individuals without laboratory-confirmed CKD had a clinical code consistent with the diagnosis. In addition, a subset of codes allowed for 1348 (0.1%) individuals receiving renal replacement therapy to be identified.

**Conclusions:**

Finding cases of CKD from primary care data using an ontological approach may have greater sensitivity than less comprehensive methods, particularly for identifying those receiving renal replacement therapy or with CKD stages 1 or 2. However, the possibility of inaccurate coding may limit the specificity of this method.

## Background

The accurate identification of CKD from primary care data facilitates the management of patients, and is vital for surveillance and research purposes [1, 2]. It may enable the timely introduction of measures to control blood pressure and reduce proteinuria, which slow CKD progression and decrease cardiovascular risk. Over the past 15 years, the diagnosis of CKD in UK primary care has improved following the introduction of a national framework for renal care and the introduction of the payment for results initiative: the Quality and Outcomes Framework (QOF) [3]. QOF data in 2014/15 indicated the adult prevalence of CKD 3–5 to be 4.1% based on clinical diagnostic coding, but it has been estimated that a further one million people may still be undiagnosed [4, 5].

Searching routinely collected computer data from primary care provides a validated method for identifying individuals with CKD [6]. However, this is not always straightforward because CKD is a heterogeneous diagnosis. The most widely used definition produced by Kidney Disease: Improving Global Outcomes (KDIGO), defines CKD as abnormalities of kidney structure or function, present for at least three months, with implications for health [7]. The role of chronicity for establishing CKD should be noted, and that a reduced estimated glomerular filtration rate (eGFR) in isolation is only diagnostic when less than 60 mL/min/1.73m^2^ (i.e. for eGFR categories 3–5). Whilst a reduced eGFR is the most important marker for CKD, the diagnostic criteria also include disorders of kidney structure and function, the most notable marker of which is proteinuria. This makes the identification of individuals with CKD stages 1 or 2 from primary care data more challenging. However, it is important not to overlook this group because they are at greater risk of all-cause and cardiovascular mortality compared to those with CKD stage 3 who do not have proteinuria [8].

### Previous studies estimating CKD prevalence in the UK

Table 1 summarises the main studies that have attempted to define the prevalence of CKD stage 3–5 in the UK, with crude estimates ranging between 4.0–8.5% [9–16]. In general, these studies only used eGFR, and did not include measures of proteinuria or clinical coding in their approach. This is particularly problematic for those with an eGFR ≥ 60 mL/min/1.73m^2^ (CKD stage 1 and 2) where a diagnosis of CKD may be missed or incorrectly assigned. Moreover, an eGFR in isolation does not allow for the identification of those with end-stage renal disease (ESRD) receiving RRT, in whom the eGFR may be misleading due to a functional kidney transplant or the dialysis process.

**Table 1:** Summary of studies estimating CKD prevalence in the UK [14–21]

Publication(s)	Cohort	Determination of CKD	Prevalence of CKD stages 3–5
Stevens 2007	130,266 patients from GP practices in Kent, Manchester and Surrey, aged 18 and over	Single eGFR (MDRD) determined from calibrated creatinines	8.5%
de Lusignan 2011, Kearns 2013	Up to 930,977 patients from practices in the Quality Improvement in CKD study (QICKD), aged 18 and over	Various methods including single and multiple eGFR readings (MDRD and CKD-EPI)	4.8–6.8%
Roderick 2011, Fraser 2015	Data from the Health Survey for England 2009–2010, involving more than 6000 participants, aged 16 and over	Single eGFR reading (MDRD and CKD-EPI) in combination with urine estimation of albuminuria	5.2–6.0%
Jameson 2014	Approximately 2.8 million individuals in the General Practice Research Database (GPRD), aged 18 and over	Two laboratory eGFR readings at least 90 days apart (MDRD) and Read diagnostic codes	5.9%
Jain 2014	Data from The Health Improvement Network (THIN) database, comprising 2,707,130 patients from 426 GP practices, aged 18 and over	2 most recent laboratory eGFR readings taken at least 7 days apart (MDRD) and Read diagnostic codes	4.0%
NCKDA 2016	Data from 911 GP practices from England and Wales, encompassing 5.2 million adults	Two eGFR calculations at least 90 days apart (MDRD), Read diagnostic codes	5.5%

*NCKDA* National CKD Audit, *GP* general practitioner, *CKD* Chronic kidney disease, *eGFR* Estimated glomerular filtration rate

The recently published year one report on the National CKD Audit included data from 911 GP practices in the UK, encompassing around 5 million adults [16]. The estimated prevalence of biochemically-confirmed CKD (defined as two eGFR results < 60 mL/min/1.73m^2^ at least 3 months apart) was 5.5%. In addition, more extensive clinical codes were used to identify CKD cases based on diagnostic coding of specific renal conditions and proteinuria (raw proteinuria values were not utilised). These codes identified a further 2.6% of the population who may have had CKD stages 1 or 2, a much lower percentage than the estimated prevalence of 6.1% in the Health Survey for England [12]. These data suggest that using primary care and diagnostic clinical codes underestimates CKD prevalence. We hypothesised that an ontological approach may identify CKD with greater sensitivity and specificity.

### The ontological approach

In information science, ontologies define concepts and their inter-relationships within a specific domain. An ontology can provide a systematic and transparent method for clinical case definition, and can also be used as a common vocabulary for researchers interested in a particular domain such as CKD. This holistic approach to case finding may estimate disease prevalence more accurately from routine data, based on definitions that are sensitive as well as specific [17, 18]. Our approach to developing an ontology involves three main steps:

- Step 1: the ontology layer, defines the concepts of relevance to the domain (CKD) and may include diagnoses, investigations, treatments and other ‘processes of care’.
- Step 2: the coding layer, identifies the most appropriate clinical codes that describe the concepts of the ontology layer. This step is specific to the coding system used in the computerised medical records.
- Step 3: the logical data extract model, represents the testing phase to ensure the data is consistent with any anticipated outputs.

In this study, our objective was to develop an ontology for identifying CKD in a primary care population and to determine the value of this approach compared to other methods that have been employed to estimate prevalence. Importantly, the concepts identified in Step 1 of the process are applicable to any healthcare coding system, so as to provide a systematic and replicable method for case finding.

## Methods

### Study cohort

Data from the Royal College of General Practitioners (RCGP) Research and Surveillance Centre (RSC) were used for the development of our method. This is a database of coded primary care data from 109 General Practitioner (GP) practices across the United Kingdom, comprising a total population of around 1.8 million patients [19]. The main clinical coding systems, or terminologies, used in primary care in the UK are Read version 2 (Read v2), Clinical Terms Version 3 (CTV3) and Systematised Nomenclature of Medicine Clinical Terms (SNOMED CT). Around 85% of general practices in the RCGP RSC use Read v2, so data were mapped to this classification for this study. The study cohort were all individuals registered with their current GP practice for at least six months prior to the index date (July 2016). Data extraction was undertaken in SQL server management studio, and statistical analysis performed using the statistical package, R.

### Laboratory-confirmed CKD

In keeping with established definitions for CKD, laboratory-confirmed CKD was based on the demonstration of either an eGFR of < 60 mL/min/1.73m^2^ or proteinuria on repeat testing at least 90 days apart. Blood test results were from samples collected in GP practices. To calculate eGFR, we applied the Chronic Kidney Disease Epidemiology Collaboration (CKD-EPI) equation to laboratory-derived creatinine readings, adjusting for ethnicity where possible [20]. To determine baseline eGFR, we used a modification of the ‘interim eGFR method’, as previously detailed by members of our group [11]. In brief, an eGFR was calculated for the most recent serum creatinine, the most recent result taken at least 90 days before, and the lowest creatinine of any results in the interim, when available. In those with at least two results available, the highest eGFR was used to determine a baseline.

Proteinuria is an important marker of CKD, particularly when the eGFR is normal or mildly reduced (≥ 60 mL/min/1.73m^2^), but may occur in the absence of kidney disease. Infections, neoplasms and bleeding involving the lower urinary tract may all give rise to ‘non-renal proteinuria’. In addition, proteinuria may be detected during an episode of acute kidney injury and not necessarily be indicative of CKD. To limit the detection of non-renal proteinuria, our method required demonstration of proteinuria on two separate occasions, at least 90 days apart. Proteinuria was defined as a urinary albumin:creatinine ratio (ACR) of ≥ 3 mg/mmol or a urinary protein:creatinine ratio (PCR) of ≥ 15 mg/mmol [21].

### Ontology and coding

A domain ontology for CKD was created in Ontology Web Language (OWL) using Protégé [22]. All concepts specific to CKD were organised according to the following categories: diagnosis (e.g. polycystic kidney disease); clinical examination finding (e.g. renal transplant palpable); investigation (e.g. dialysis adequacy test); treatment (e.g. dialysis); procedure (e.g. arteriovenous fistula formation); complication of care (e.g. renal transplant rejection); and process of care (e.g. CKD annual review). Concepts included in the ontology were consistent with the KDIGO criteria for the definition of CKD, excluding structural abnormalities not associated with primary kidney damage (such as renal tumours or a single kidney) [7]. The ontology, applicable to any healthcare coding system, has been published online and can be accessed using the following link [23]: http://bioportal.bioontology.org/ontologies/CKDO.

We then identified concepts from the CKD ontology using the Read v2 hierarchy and NHS Read Code Browser Version 20.0. We identified around 1250 candidate codes from a total of over 80,000. These were reviewed by a panel of three consultant nephrologists (PAS, RJS, HG), as well as clinical informatics experts (SDL, JW), who agreed upon the final 605 Read codes for inclusion. 158 of these codes implied that a person had received RRT and were used to identify individuals receiving dialysis or with a renal transplant.

## Results

**Fig. 1:**
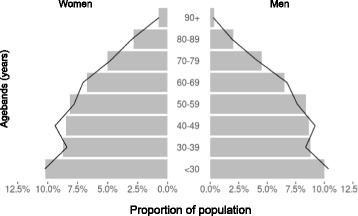
Age-sex profile of the study cohort. The black line represents age-sex distribution of the general population in England and Wales (from 2011 National Census data)

**Fig. 2:**
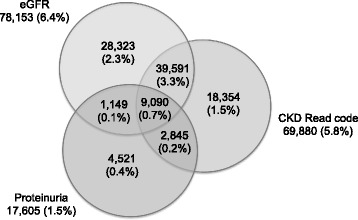
Venn diagram demonstrating the number individuals identified with chronic kidney disease (CKD). Three main approaches were utilised: an estimated glomerular filtration rate of < 60 mL/min based on at least 2 blood tests taken at least 90 days apart; proteinuria demonstrated on two urine tests taken at least 90 days apart; a Read code consistent with a diagnosis of CKD. Data shown are number of individuals (percentage of population)

The study population comprised 1,213,679 individuals, with an age-sex distribution similar to census data from England and Wales (Fig. 1) [24]. 617,352 (50.9%) were female and the median age was 47 years (IQR 30). 787,032 (64.8%) were identified as being of white ethnicity, 71,493 (5.9%) of Asian ethnicity and 37,649 (3.1%) of black ethnicity. The ethnicity was unknown in 291,499 (24.0%) of the cohort. A summary of the main approaches used to identify CKD is shown in Fig. 2.

### Identification of CKD using eGFR

837,396 individuals (69.0%) had at least one creatinine result available on the database. The median number of months since the most recent creatinine test was 12 (IQR 4–33). 733,749 (60.5%) individuals had a creatinine result within the past 5 years, and 570,409 (47.0%) within the past 2 years. 620,375 (74.1%) of individuals with at least one creatinine result had a second result at least 90 days prior to the first, allowing chronicity to be established. 78,153 individuals had an eGFR < 60 mL/min/1.73m^2^, giving a prevalence of CKD stage 3–5 of 6.4% (Table 2).

**Table 2:** Summary of the number of CKD cases by KDIGO stage, using the three main approaches

	Method for identifying CKD		
CKD stage	eGFR	eGFR + Proteinuria	eGFR + Proteinuria + CKD Code
Unknown	–	41 (< 0.1%)	2296 (0.2%)
1	–	2211 (0.2%)	4481 (0.4%)
2	–	5114 (0.4%)	19,763 (1.6%)
3A	52,662 (4.3%)	52,662 (4.3%)	52,487 (4.3%)
3B	19,396 (1.6%)	19,396 (1.6%)	19,196 (1.6%)
4	5023 (0.4%)	5023 (0.4%)	4865 (0.4%)
5	1072 (0.1%)	1072 (0.1%)	587 (< 0.1%)
5 - RRT	–	–	1348 (0.1%)
CKD 3–5	78,153 (6.4%)	78,153 (6.4%)	78,483 (6.5%)
CKD 1–5	78,153 (6.4%)	85,519 (7.0%)	105,023 (8.7%)

Data are shown by number of individuals (percentage of study cohort). Abbreviations: *CKD* Chronic kidney disease, *eGFR* Estimated glomerular filtration rate, *RRT* Renal replacement therapy

### Identification of CKD using proteinuria estimations

17,605 (1.5%) of the study cohort were found to have proteinuria: 7325 of these individuals had CKD stages 1–2, as determined by eGFR; 3978 had CKD stage 3A; 3736 had CKD stage 3B; 1905 had CKD stage 4; and 620 had CKD stage 5 (Table 2).

### Identification of CKD using ontology-derived read codes

69,880 individuals (5.7% of the cohort) had a Read code consistent with a diagnosis of CKD. 48,681 (62.3%) of the 78,153 with CKD stages 3–5, as determined by eGFR, had a CKD Read code. In those identified with CKD using both eGFR and proteinuria (laboratory-confirmed CKD), 51,526 (60.5%) out of 85,119 had a CKD Read code. In those without laboratory-confirmed CKD, there were 18,354 individuals with a CKD Read code, the majority of whom had CKD stage 1 or 2 based on eGFR (Table 2). RRT codes identified a total of 1348 individuals either on dialysis or with a functioning renal transplant that were not identifiable using laboratory measures.

## Discussion

In this large cross-sectional study, we have described a comprehensive strategy for identifying CKD from primary care data in the UK. It is the first such study to combine estimations of both GFR and proteinuria with clinical coding using an ontological method. This case-finding approach found the prevalence of CKD stages 1–5 to be 8.7% in a population of over 1.2 million patients, which is lower than the observed prevalence of 13–14% in the Health Survey for England [13]. Using eGFR in isolation, the prevalence of CKD stages 3–5 was 6.4%, which is in keeping with previous reports [9–16].

Proteinuria estimations detected 0.6% of the cohort to have CKD who did not meet the diagnostic criteria based on eGFR in isolation, and clinical coding identified a further 1.6%. The majority of these individuals were classified as having CKD stage 1 or 2. This is of importance given that mild-to moderate CKD is predominantly managed in the community, and the crucial role of general practice for identifying these patients and treating them appropriately [3]. It is particularly valuable to identify those with proteinuria, because these individuals are at higher mortality risk independent of eGFR [8].

This study has also confirmed the limitations of using clinical coding in isolation for case finding. CKD Read codes were present in 62% of those with an eGFR < 60 mL/min/1.73m^2^, affirming the possibility that a significant proportion of CKD in the community may be unrecognised. Our ontological approach did not improve upon the 70% of people identified using only QOF-derived codes in the recently published National CKD Audit [16]. We also found that 26% of individuals with a CKD Read code did not meet the criteria for CKD on the basis of laboratory testing alone. Even allowing for limitations of our method, and for the fact that a normal eGFR does not preclude a diagnosis of CKD, it could be that a significant proportion of individuals are incorrectly coded. To ascertain this would require analysis on a case-by-case basis.

An advantage to our method was the identification of people with ESRD receiving RRT. Using a subset of CKD Read codes, the ontology enabled 1348 individuals to be identified who had received either a renal transplant or dialysis. Although this group represents only 1.6% of those with laboratory-confirmed CKD, these patients are at particularly high-risk of complications and to our knowledge, this is the first description of a method to identify them from routinely collected primary care data. However, it should be noted that the prevalence of ESRD in this cohort was 1110 per million population, 18% higher than the prevalence reported in the latest UK Renal Registry Report (941 per million population) [25]. Whilst it is possible that this could represent a higher rate of ESRD in this cohort, it may also be indicative of some of the limitations with coding discussed below.

### Limitations

We have described a comprehensive method for identifying CKD from primary care coding that has been applied to a large cohort. However, there are inevitable limitations that come with using routinely collected data, including missing and incorrectly coded information. It is only possible to identify CKD in patients who have visited their GP or had a blood test taken. Furthermore, the busy and high turnover nature of General Practice results in the unavoidable reality that aspects of the medical history may not be coded, including historical diagnoses that may have preceded computerised medical records. Even with fastidious coding, the Read code hierarchy lacks sufficient granularity in some areas to accurately determine all cases of CKD. Whilst our ontology was created to be as comprehensive as possible, it is limited by the fact that some concepts are not present in the Read code terminology, whilst other are non-specific or do not sufficiently differentiate between acute and chronic. Also of note, the NHS in England is to change from using Read codes to using the SNOMED CT by April 2018, and Read codes had stopped being updated at the time of this study [26, 27].

Finally, we have used two logical models to derive eGFR and proteinuria based on multiple readings taken over at least 90 days. Whilst these methods improve upon the use of single readings, they do not completely overcome the confounding issue of AKI, the limitations of the tests, and fluctuations in disease states. Additionally, missing ethnicity coding will underestimate eGFR in those of black ethnicity, and inter-laboratory variation in the creatinine assay may influence prevalence rates. It should also be noted that the database blood results are from samples collected in GP practices, and results taken elsewhere will not be accounted for.

## Conclusions

Ontological methods are well established in information science and may facilitate the accurate case finding of CKD from primary care data. We have published a novel ontology that sets out a broad range of concepts relevant to a diagnosis of CKD. These concepts formed the basis of a multifaceted approach to case finding, combining laboratory indices with clinical codes. This method may identify CKD with greater sensitivity than existing methods, particularly when it comes to identifying those with ESRD receiving RRT or those with CKD 1 or 2. However, notwithstanding that the iterative ontological process is intended to exclude ambiguous codes, the quality of routine data may limit the specificity of this method. Future studies to validate CKD coding in a subset of individuals would be of value.
